# Dr. Alonzo P. Beale

**Published:** 1893-02

**Authors:** 


### Obituary.

### DR. ALONZO P. BEALE.

DR. ALONZO P. BEALE died in Germantown, Philadelphia, Pa., January 4, 1893. His health for two years had been a source of great anxiety to his friends, but not until inflammation of the lungs became manifest did they deem the end so near. He leaves a wife, two children, and a host of friends to mourn his early death.

Dr. Beale was born in Philadelphia, February 7, 1857, and was therefore thirty-six years of age. He studied dentistry with his father, and, after taking two full courses at the Pennsylvania College of Dental Surgery, graduated from that institution in March, 1879. For about thirteen years he has been connected with his Alma Mater as Demonstrator of Mechanical Dentistry, giving each year, very acceptably to the Faculty and the class, a course of lectures on Dental Prosthesis, these supplementing the lectures from the chair of Prosthetic Dentistry. As a teacher, Dr. Beale was quiet, unassuming, and thorough, and enjoyed the respect and affection of all with whom he came in contact. Rarely do we have to record the loss of one so young, who leaves so great a blank among his circle of associates.

C. N. P.

---

At a meeting of the Faculty of the Pennsylvania College of Dental Surgery, held January 9, 1893, the following resolutions were adopted:

*Resolved,* That in the death of Dr. Alonzo P. Beale, who for thirteen years filled the position of Demonstrator and Lecturer in the Pennsylvania College of Dental Surgery, the Faculty recognizes the loss not only of a teacher of rare ability, but of a man whose admirable personal qualities endeared him to all with whom he was associated.

*Resolved,* That a formal expression of the heartfelt sympathy of the members of this Faculty be conveyed to the family of their deceased coadjutor, with the assurance that their profound regret at his untimely loss will be fully shared in by every student and alumnus of the institution.

(Signed) Henry Leffmann,
*Secretary of Faculty.*

